# Treatment of *H. pylori* infection and gastric ulcer: Need for novel Pharmaceutical formulation

**DOI:** 10.1016/j.heliyon.2023.e20406

**Published:** 2023-09-24

**Authors:** Ashutosh Gupta, Shiran Shetty, Srinivas Mutalik, Raghu Chandrashekar H, Nandakumar K, Elizabeth Mary Mathew, Abhishek Jha, Brahmeshwar Mishra, Siddheesh Rajpurohit, Gundawar Ravi, Moumita Saha, Sudheer Moorkoth

**Affiliations:** a Department of Pharmaceutical Quality Assurance, Manipal College of Pharmaceutical Sciences, Manipal Academy of Higher Education, Manipal 576104, Karnataka, India; b Department of Gastroenterology and Hepatology, Kasturba Medical College, Manipal Academy of Higher Education, Manipal 576104, Karnataka, India; c Department of Pharmaceutics, Manipal College of Pharmaceutical Sciences, Manipal Academy of Higher Education, Manipal 576104, Karnataka, India; d Department of Pharmaceutical Biotechnology, Manipal College of Pharmaceutical Sciences, Manipal Academy of Higher Education, Manipal 576104, Karnataka, India; e Department of Pharmacology, Manipal College of Pharmaceutical Sciences, Manipal Academy of Higher Education, Manipal 576104, Karnataka, India; f School of Pharmacy, Faculty of Health Sciences, University of Botswana, Gaborone, Botswana; g Department of Pharmaceutical Engineering and Technology, Indian Institute of Technology (BHU), Varanasi 221005, Uttar Pradesh, India

**Keywords:** *Helicobacter pylori*, Peptic ulcer disease, Antibiotic resistance, GRDDS, Nanomedicine

## Abstract

Peptic ulcer disease (PUD) is one of the most prevalent gastro intestinal disorder which often leads to painful sores in the stomach lining and intestinal bleeding. Untreated *Helicobacter pylori* (*H. pylori)* infection is one of the major reasons for chronic PUD which, if left untreated, may also result in gastric cancer. Treatment of *H. pylori* is always a challenge to the treating doctor because of the poor bioavailability of the drug at the inner layers of gastric mucosa where the bacteria resides. This results in ineffective therapy and antibiotic resistance. Current treatment regimens available for gastric ulcer and *H. pylori* infection uses a combination of multiple antimicrobial agents, proton pump inhibitors (PPIs), H2-receptor antagonists, dual therapy, triple therapy, quadruple therapy and sequential therapy. This polypharmacy approach leads to patient noncompliance during long term therapy. Management of *H. pylori* induced gastric ulcer is a burning issue that necessitates alternative treatment options. Novel formulation strategies such as extended-release gastro retentive drug delivery systems (GRDDS) and nanoformulations have the potential to overcome the current bioavailability challenges. This review discusses the current status of *H. pylori* treatment, their limitations and the formulation strategies to overcome these shortcomings. Authors propose here an innovative strategy to improve the *H. pylori* eradication efficiency.

## Graphical abstract

**Figure:**
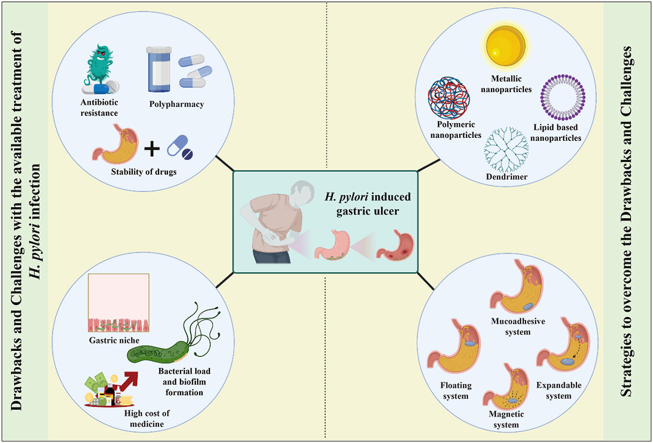

## 1 Introduction

**Fig. 1:**
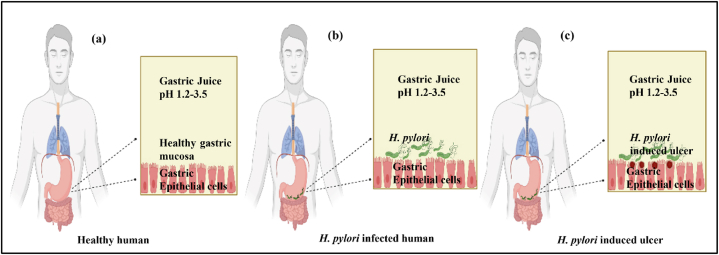
Pictorial representation of *H. pylori* infection and Peptic Ulcer Disease (a) Stomach of healthy human (b) *H. pylori* infected stomach (c) Stomach of *H. pylori* induced ulcer.

*Helicobacter pylori (H. pylori)* is a microaerophilic, spiral-shaped, multi-flagellate gram-negative bacterium found in the human gastric mucosa, which infects nearly 50% of the population in the world [1]. Barry Marshall and Robin Warren first identified *H. pylori* in 1982. In 1989, the bacterium formerly known as *Campylobacter pylori* (*C.pyloridis*) was reclassified as *H. pylori* [2,3]. The proof of concept that *H. pylori* infection causes gastritis was established through self-experiments involving the ingestion of a bacterial broth, followed by subsequent recovery from gastritis upon eradication of *H. pylori*, thus verifying Koch's postulates [4]. Clinical investigations have substantiated Koch's postulates, which assert that an illness must be attributed to a specific pathogen and can be effectively cured by eliminating the said pathogen from the host [5]. Marshall and Warren were awarded the Nobel Prize in physiology for their ground-breaking discovery, which demonstrated that the permanent healing of peptic ulcers could be achieved through the eradication of *H. pylori*. The primary routes of this infection are oral-oral and oral-fecal interactions. Dirty water can also be a source of this infection in developing countries. The ability to culture *H. pylori* from infected individuals' stool, dung, and saliva suggests the possibility of its transmission through these routes [6]. A pictorial representation of a healthy and the *H. pylori* infected human stomach is provided in Fig. 1a and b, respectively.

Untreated *H. pylori* infection lead to many disease conditions related to GI tract including gastritis, non-ulcer dyspepsia, peptic ulcer disease, antral polyp, gastric adenocarcinoma, MALT lymphoma, follicular esophagitis, gastroesophageal reflux disease, esophageal adenocarcinoma, diffuse duodenal nodular lymphoid hyperplasia, immunoproliferative small intestinal disease, and inflammatory bowel disease. Some non-GI tract associations of *H. pylori* infection are hepatobiliary diseases, pancreatic disease, cardiovascular disorders, endocrine and metabolic disorders, dermatological disorders, haematological disorders, neurological disorders and ophthalmic disorders [7]. A pictorial representation is provided in Fig. 1c for *the H. pylori* induced gastric ulcer. This infection is associated with symptoms like abdominal pain, loss of hunger, sickness, and lethal complications like gastrointestinal bleeding. Extra-intestinal symptoms of *H. pylori* include but are not limited to: short stature, immune thrombocytopenic purpura, refractory iron deficiency anaemia (IDA), vitamin B12 insufficiency, eye diseases, skin disorders, respiratory disorders, and mental health issues [8,9]. Various diagnostic approaches are available for the infection including histological examination and rapid urease test on gastric mucosal biopsies, urea breath tests etc. The selection of one method over the other depends on several factors, such as convenience, cost, age of patients etc. [10]. Peptic ulcer, which affect 5–10% of the population are acid-induced lesions of the digestive system, characterised by denuded mucosa with the defect spreading into the submucosa or muscularis propria. *H. pylori* infection has been reported as a major cause for peptic ulcer disease among the various other reasons like consumption of alcohol, tobacco, cigarette, non-steroidal anti-inflammatory drugs (NSAIDs), Zollinger-Ellison syndrome and idiopathic formation [11]. The untreated peptic ulcer may be fatal and short-term complications includes hypovolemia, shock, sepsis and gastrocolic fistula formation.

*H. pylori* demonstrate specialized adaptations enabling it to successfully inhabit the stomach's mucus layer, an environment known for its challenging conditions. This colonization is driven by a range of essential processes, including motility, urease production, adhesion, and various other mechanisms. *H. pylori* after entering into the human stomach pass to the gastric lumen. In the lumen, the urease gene cluster present in the bacterium get activated in the presence of nickel cofactor. This regulates proton-gated urea channel and allow urea entry only in acidic environment. When pH is lower than 5, there is a rapid entry of urea into the bacterium which in the presence of the urease enzyme is converted to ammonia and carbon dioxide. Ammonia further produces ammonium hydroxide in the presence of water. The ammonium hydroxide neutralizes the surrounding environment of the *H. pylori* and protect it from the acidic surroundings [12,13]. After surviving the acidic conditions of the lumen, *H. pylori* travel through the gastric mucosal layer (pH value is close to 7) with the help of flagella [14,15]. Upon infection, the surface adhesins of *H. pylori* attaches to the gastric mucosa, which primarily interact with mucin 5 (MUC5AC). The elevated production of MUC5AC can be a potential mechanism for promoting bacterial adherence [16]. Adhesins contain toxins like neutrophil activating protein (NAP), which stimulate the production of free-radicals and lead to local tissue damage, release of IL-8, TNF-α etc., thereby resulting in chronic gastritis [13]. *H. pylori* infection lead to an increased synthesis of certain inflammatory cytokines and stop the synthesis of prostaglandin, which may contribute to inflammation and ulcer formation [[17], [18], [19], [20]]. The damage in the stomach mucosal lining in the corpus-predominant area of stomach leads to sipping of stomach acid in the epithelial cells, further inflaming the area and causing sore formation or ulcer. *H. pylori* can also interfere with the normal mechanisms involved in repairing damaged stomach tissue, thereby delaying the healing of existing ulcers or promoting the development of new ones [14,15]. Also, the VacA proteins released from *H. pylori* disrupts the balance of cell proliferation and death by affecting the genes that regulate the cell cycle leading to cell apoptosis and ultimately gastric cancer [7,13].

Greater than 90% of *H. pylori* is present in the extracellular space of the mucosal layer of the stomach. A low population of the bacteria reside in the intracellular vacuoles and migrate to the extracellular space to repopulate the bacteria in the mucosal layer [21]. While residing in the intracellular space the bacteria does not undergo cell replication and thus the clinical significance of the bacterium's invasiveness is negligible [22]. However, these dormant bacteria can repopulate after coming back to the extracellular space when the antibiotic concentration is less [23]. The ability of the organism to transfer between intra and extracellular spaces is a major reason for the ineffectiveness of current treatment regimen resulting in incomplete cure, bacterial resistance, which prevents eradication of the organism. Novel formulations having extended-release ability and possessing both localized and systemic action should be a strategy for effective eradication of *H. pylori*.

Most of *the H. pylori* cases occur in middle- and low-income nations*.* [[24], [25], [26]]. The infection is more prevalent in Africa and East Asian nations. The reported seroprevalence rate are 60% in South Korea, 44% in China, and 39% in Japan. The prevalence of *H. pylori* in Africa ranged from 87% in South Africa [27] to 91% in Nigeria [28]. The incidence of *H. pylori* infection in Middle East and North African nations (MENA) was 40%. However, countries like Egypt (72–88%), Iran (82%), Israel (40–75%), Lebanon (21%), Libya (76%), Oman (68.4%), Saudi Arabia (28–70%), and Tunisia (82.7%) [29] had higher prevalence. There is a prevalence of 69.4% reported in South America. The frequency of *H. pylori* is modest in developed nations like North America (37.1%), Europe (20–40%), and Australia (16–30%). The incidence of *H. pylori* infection was 42.7% in females and 46.3% in men. The age-wise prevalence statistics reveal that adults are more vulnerable than children. According to reports, prevalence among minors was 32.6%, while in the age groups of 18–30 years it was 40.5% and in the age group of 31–50 years, it was 43.6% [30,31]. *H. pylori* is the leading cause of peptic ulcer. Greater than 11% of the global population are suffering from peptic ulcer induced by prolong *H. pylori* infection. A study revealed 70–95% co-relation between gastric ulcer and *H. pylori* infection worldwide [4].

Treatment of *H. pylori* infection is always a challenge for the treating doctor because of the ineffectiveness of the available treatment options. Currently approved treatment for combating *H. pylori* is a combination of two or more antibiotics and anti-ulcer drug in the traditional delivery mode. This strategyfail in the eradication of *H. pylori* infection because of the insufficient reach of antibiotics to the inner mucosal layer of the stomach lining where the bacteria reside [[32], [33], [34], [35], [36]]. This often results in treatment failure due to antibiotic resistance, low medication absorption and poor patient compliance due to polypharmacy [21,23,37]. However, the above challenges can be effectively addressed by utilizing a novel drug delivery method. Since *H. pylori* has the ability to reside extracellularly as well as inside intracellular vacuoles, a formulation that can deliver the drug over a long period locally as well as systemically will be able to eradicate the organism from the system [23]. This article discusses the existing therapeutic regimes, challenges associated with effectively treating *H. pylori*, and novel strategies to eradicate *H. pylori* infection.

## 2 Available therapy options for *H. pylori*-induced gastric ulcer and their drawbacks

A combination of antibiotics and gastric acid-suppressing medications such as H_2_ -antagonists and proton pump inhibitors (PPIs) are generally the preferred choice for the *H. pylori* eradication. A detailed list of currently used drugs for treating *H. pylori* infection according to their therapeutic classes is mentioned in Table 1 [[38], [39], [40]]. The increase in antibiotic resistance has become a major obstacle in eradicating *H. pylori* [41]. Different approaches and drug combinations are employed to reduce this issue, but doing so has increased the number of medicines that must be administered, which in-turn reduced patient compliance [42]. H_2_ antagonist reduces acid secretion but does not affect *H. pylori* bacteria [43]. In contrast, PPIs are preferred due to their bacteriostatic activity against *H. pylori*. The adverse effects of PPIs can be separated into two categories: acid inhibition-related and non-acid inhibition-related. The majority of acid inhibition-related side effects (increased gastric pH, hypochlorhydria and in some cases achlorhydria) occur during long-term use of PPI, but non-acid inhibition-related adverse effects occur in both long-term and short-term PPI users [44]. In addition, long term treatments have a considerable impact on the gut flora resulting in a variety of digestive diseases and metabolic abnormalities, including dysbiosis of the gut microbiota [45].

**Table 1:** A complete list of medications used to treat *H. pylori* infection and the reported bioavailability.

Sl. No.	Therapeutic class	Mechanism	Drugs	Bioavailability after oral administration
**1.**	β lactams	Prevent the synthesis of peptidoglycan layer of bacterial cell walls.	Amoxicillin	70% [46]
			Penicillin V	60–70% [47]
			Ampicillin	39–54% [48]
**2.**	Macrolides	Attach to the 50s ribosomal subunit and prevent the production of proteins.	Clarithromycin	55% [49]
			Erythromycin	30–65% [50]
			Azithromycin	37% [51]
**3.**	Tetracyclines	Prevents the production of protein by binding to the 30s ribosomal subunit	Tetracycline	60–80%^a^
			Doxycycline	73–95%^a^
**4.**	Nitroimidazoles	Inhibit protein production by binding with DNA and causing a loss of helical DNA structure and strand breakage.	Metronidazole	90%^a^
			Tinidazole	99% [52]
**5.**	Quinolones	Inhibit replication of bacterial DNA by blocking the bacterial DNA gyrase	Ciprofloxacin	70%^a^
			Norfloxacin	85% [53]
			Levofloxacin	99%^a^
			Moxifloxacin	90%^a^
**6.**	Ansamycins	Inhibition of DNA-dependent RNA polymerase	Rifabutin	20% [54]
			Rifamycin	68% [55]
**7.**	H_2_ Antagonists	Bind on histamine type 2 receptor and block the acid secretion	Cimetidine	60%^a^
**8..**	PPIs	Bind to H+/K + ATP pump to stop acid secretion.	Omeprazole	30–40%^a^
			Pantoprazole	77%^a^
			Lansoprazole	80–90%^a^
			Esomeprazole	90%^a^
			Rabeprazole	52%^a^

a Based on the PubChem data. H2 antagonist: Histamine H2-receptor antagonists, PPI: proton pump inhibitors, DNA: Deoxyribonucleic acid.

### 2.1 First-line treatment

The patient's history of antibiotic use should be gathered before deciding on an *H. pylori* treatment strategy. For the first 10–14 days of therapy, patients with an *H. pylori* infection are given a combination of two antibiotics along with a PPI. The antibiotic of choice in the first-line treatment are amoxicillin, clarithromycin, and metronidazole. Clarithromycin based therapy is safe and effective, but it is important that before this therapy, the patient should not have previous exposure to clarithromycin and other macrolide class of drugs. It is possible that previous exposure to antibiotics can lead to drug resistance and failure of the therapy. Lee et al. reported that clarithromycin-based triple therapy had a 90% efficacy rate, but it has currently reduced to 70–80% efficacy rate [56]. Bismuth-based quadruple therapy is the alternate option for the resistance in clarithromycin-based therapy. A clinical study had compared the efficacy of cclarithromycin and bismuth-based therapies in infected patients. Results of this study indicate that bismuth based quadruple therapy showed 93.3% eradication, and clarithromycin based could achieve only 69.6% eradication [57,58]. Quadruple therapy may be an effective treatment option for *H. pylori* infection; but, the availability of bismuth and associated complications is a cause of concern [59].

### 2.2 Second-line treatment

A second-line therapy is administered if the first treatment fails. In this therapy levofloxacin, amoxicillin and PPI triple regimen is used. It is reported that 10 days of treatment had resulted in 84% cure in patients [60]. Bismuth-containing quadruple therapy is also used as the second-line treatment. Gisbert et al. reported adverse events in patients with second-line therapy, like metallic taste, nausea, vomiting, abdominal pain, and diarrhea [61]. Vakil et al. reported that the prolonged course of the second-line treatment and premature discontinuation of the treatment can lead to antibiotic resistance and other side effects [62]. If first-line treatment with clarithromycin does not work, the Maastricht V/Florence consensus report suggests using either a colloidal bismuth pectin–containing quadruple therapy or a fluoroquinolone–containing triple or quadruple therapy [63]. Nista et al. reported that levofloxacin, amoxicillin, and rabeprazole exhibit 94% eradication of *H. pylori* bacteria while levofloxacin, tinidazole, and rabeprazole depicted 90% elimination for *H. pylori* bacteria [64].

### 2.3 Third-line treatment

It is the rescue therapy for *H. pylori* infection. Third-line treatment is used when the other two therapies have failed, or resistance has developed to the other therapy. Third-line therapy is rifabutin-based therapy. The treatment regimen is rifabutin, levofloxacin, amoxicillin, and PPI. The eradication rate of this therapy ranges from 75 to 90% [[65], [66], [67]]. Rifabutin, is a derivative of rifampicin, can induce a severe but rare myelotoxicity that appears to be dose-dependent, and its widespread use may cause drug resistance in patients with tuberculosis. Rifabutin should only be used as a "rescue" therapy if other antibiotics have failed in eradicating *H. pylori* [68]. Sitafloxacin is a new quinolone derivative with improved action against *H. pylori* with gyrA mutations as well as imparting a higher efficacy than levofloxacin-based treatment as a third-line therapy [69].

Suzuki et al. reported that Rifaximin, in comparison with rifabutin, is less absorbed into the bloodstream and hence nearly free of side effects and possess a better bioavailability in the gastrointestinal tract [70]. Tursi et al. examined the effectiveness of Levofloxacin-containing third-line treatment for 10 days on 119 patients in Southern Italy. Overall, 24.37% of individuals suffered side effects, with 1.68% having been affected by severe side effects which led to patient drop-out from the trial [71].

## 3 Drawbacks of the current treatment regimens

The major drawback of the available treatment is antibiotic resistance. This is mainly due to the ineffective reach of these antibiotics to the organism since it resides in the inner layer of the intestinal mucosa. The bacteria are also resistant to the highly acidic environment of the stomach. Penicillin-allergic patients cannot be administered amoxicillin-based treatment regimen. Another drawback is the use of polypharmacy, where many pills are to be taken together, which leads to a lot of inconvenience and omission/missing of dosage by patients. Usage of multiple antibiotics can affect the gut microbiome. It also adds to the cost of treatment [59]. The unavailability of bismuth coupled with side effects such as metallic taste, nausea, myalgia/arthralgia, and diarrhea are significant problems associated with the bismuth-based therapy [42]. Rifabutin based therapy has side effects such as leukopenia and thrombocytopenia, with myelotoxicity. This can also develop resistance against mmycobacterium avium in HIV-infected patients [68]. No treatment of *H. pylori* is 100% effective and after the completion of treatment, the chance of reinfection is still a problem [72].

## 4 Challenges associated with the available treatment of *H. pylori*-induced gastric ulcer

### 4.1 Antibiotic resistance

The primary cause of the treatment failure against *H. pylori* infection is antibiotic resistance, which arises due to the irrational use of antibiotics and insufficient bioavailability [73]. Antibiotics generally used for treating *H. pylori* are amoxicillin (AMX), metronidazole (MET), clarithromycin (CLR), tetracycline (TET), and levofloxacin (LEV). Earlier researchers have studied the resistance of these antibiotics. The primary resistance rates to *H. pylori* for AMX, CLR, MET, LEV, and TET were reported to be 15.0%, 34.1–55.2%, 69.4–71.3%, 18.4–27.9%, and 17.9%, respectively. The secondary resistance rates reported for these antibiotics were 9.5%, 74.9%, 61.5%, 45.7%, and 23.5%, respectively [73]. The study also suggested that a single antibiotic is ineffective in eradicating *H. pylori* and leads to resistance. To solve this problem, a combination of antibiotics and PPI are used [[74], [75], [76], [77]]. Increasing the dose and the period of therapy with acid suppression is a common strategy for overcoming metronidazole resistance. They concluded that the highest rates of cure are achieved with greater doses and longer therapy periods. Salvage treatment regimens like bismuth or furazolidone quadruple treatment are being utilized after other treatment regimens are unsuccessful [78].

### 4.2 Patient-related challenges

Patient noncompliance is the major challenge in treating *H. pylori*. This is because the patient must take more than ten different medications. According to one survey, patients who took less than 60% of medication had only a 30% efficacy rate and also resulted in antibiotic resistance [79,80]. The results of a study aiming to find variables affecting the success of triple treatment indicated that noncompliance is the leading reason of *H. pylori* eradication failure. Age, gender, illness type of treatment, length of treatment, and bismuth dosage had no influence on the eradication rate [81]. In order to eradicate *H. pylori*, patients often use antibiotics and acid-suppressing drugs for a period of 1–2 weeks. Patients may have trouble sticking to their treatment plan because of unpleasant taste, side effects and the complexity of their medication. Nonadherence to *H. pylori* medication may result in treatment failure, recurrent infection, and antibiotic resistance [82].

### 4.3 Rapid metabolism of PPIs

PPI is metabolized by cytochrome P450, which is made of different isoenzymes. The isoenzyme used in the metabolism of PPI is CYP2C19, which decreases the potency of PPI. To overcome this problem, it is recommended to increase the dose of PPI, [83,84]. Ormeci et al. reported that Cytochrome P450 2C19 (CYP2C19) variant is important for PPI metabolism. Therefore, CYP2C19 genotyping before therapy may make it easier to determine the ideal PPI dose to enhance the therapeutic result [85]. Morino et al. conducted a meta-analysis to find out the effect of cytochrome P450 2C19 genotype on the PPI metabolism for *H. pylori* treatment. In the fixed-effects model, the meta-analysis revealed that the relative risk of unsuccessful eradication in CYP2C19 extensive metabolism compared with intermediate metabolism and poor metabolism was 1.21 (95% CI: 1.06–1.39, *p* < 0.006) and 1.57 (95% CI: 1.27–1.94, *p* < 0.001), respectively. The cure rate of the eradication regimens involving omeprazole and lansoprazole in combination varied amongst CYP2C19 genotypes, in contrast to the regimens combining rabeprazole and esomeprazole (*p* < 0.05) [86].

### 4.4 Stability of drugs in acidic gastric environment

Many drugs degrade in gastric fluid due to hydrolysis and enzymatic action. Another issue plaguing this class of drugs is the ionization in the acidic GI environment which prevents the permeability [87]. Erah et al. evaluated the stability of amoxicillin, clarithromycin, and metronidazole in gastric juice in the presence of omeprazole. Results show that omeprazole is expected to increase the chemical stability of amoxicillin and clarithromycin in gastric juice. While amoxicillin and metronidazole are sufficiently stable at this pH to maintain an antibiotic concentration in the stomach, clarithromycin degrades quickly at the typical gastric pH (1.0–2.0) [88].

### 4.5 Morphology of bacteria and biofilm formation

Bacteria change their morphology, which protects them from eradication. In general, bacteria shrink and transform into tiny spherical entities with modified gene expression, and molecular synthesis. *H. pylori* create a neutral environment surrounding it in the mucosal layer via its adaptive mechanisms and thereby, protects it from gastric acid [89]. The bacteria's morphology will have an effect on its capacity to move, aggregate, and form biofilms, as well as its resistance to potentially harmful environmental influences. Peptidoglycan and cytoskeleton rearrangements alter the shape and viability of *H. pylori* [90]. Biofilm provides physical and chemical diffusion barriers to antimicrobial penetration [91]. It is composed of living microorganisms, a variety of nucleic acids (extracellular DNA from bacteria), self-produced extracellular polymeric materials (EPS), such as polysaccharides, and proteins [92]. Studies showed that *H. pylori* bacteria can form biofilm, including the clinical, slandered, and mouse adapted strains [[74], [75], [76], [77]]. Hathroubi et al., reported that these structures might contribute to the pathogen's persistence, adaptability, or resistance to antimicrobial therapy in the host or environment [93]. It is also reported that *H. pylori* changes its phenotype during biofilm development, which reduces its susceptibility to antibiotics. However, extracellular protease therapy can somewhat improve this resistance [94,95]. Additionally, *H. pylori* has the ability to survive in both aerobic and anaerobic conditions and also live both extracellularly and intracellularly. It can stay in the intracellular vacuoles and can repopulate after coming in the extracellular space when the antibiotic concentration is less [23]. This is a major challenge that results in antibiotic resistance.

### 4.6 Gastric niche

Gastric niche protects the bacteria from the gastric juice and allows the colonization of other bacteria. *H. pylori* quickly adapts to the gastric environment. It produces ammonia and HCO_3_ via urease and α-carbonic anhydrase, to produce ammonia, making a protective natural layer outside the bacteria. A higher local pH makes it easier for bacteria to pass through stomach fluid [[96], [97], [98]]. Studies prove that the gastric niche stimulates flagellar proteins and flagellin to further protect *H. pylori* against harsh circumstances and acid exposure, thereby improving *H. pylori* motility and help to accumulate at a higher density on the stomach epithelium, resulting in a larger inflammatory response [[99], [100], [101]].

### 4.7 Bacterial load

Bacterial load is also the reason for increased acute mucosal damage and for failure in *H. pylori* therapy. The high density of bacteria can increase the mucosal inflammatory cytokine responses, thus decreasing the immune response. Eradication of *H. pylori* can decrease the inflammation in gastric mucosa and reduce the bacterial load [102,103]. Lia et al. reported the relation between the *H. pylori* bacterial load and ulcer curing rates in patients with active duodenal ulcers. Few studies showed that in the groups with mild, moderate, and marked *H. pylori* infection, the success rates of *H. pylori* treatment were 88.9%, 94.3%, and 69.7%, and that of ulcer healing were 88.0%, 94.3%, and 63.6% respectively. The statistical analysis showed that both the eradication rate and ulcer curing rate had a strong association with pretreatment *H. pylori* density (*p* = 0.013/0.006 and 0.002/<0.001, respectively) [104]. Belda et al. has reported the link between bacterial burden, morbidity, and the cagA protein (*H. pylori* virulence factor). Result shows that both in the antrum and corpus, the bacterial burden in patients with cagA positive strain was higher than in those without it (*p* < 0.01). A statistically significant link existed between the intake of proton pump inhibitors and cagA [105].

## 5 Strategies to overcome the drawbacks: need for novel formulation

The above discussed challenges make the treatment of *H. pylori* and gastric ulcer ineffective. Many antibiotics show low *in vivo* efficacy due to the failure of the drug to reach the target site of action. *H. pylori* resides in the inner layer of stomach lining, where the bioavailability of a drug is low, and the plasma concentration drops based on the half-life (t_1/2_) of the drug [106]. To maintain an adequate level of the drug, it is necessary to re-administer the drug at specific intervals. Controlled drug delivery is the one approach for regulating drug bioavailability and to increase a drug's efficacy. There are many directed delivery approaches. One of them is the gastro-retentive drug delivery system (GRDDS), which aims to retain the formulation in the stomach for longer period of time and release the medicine sustainably. GRDDS also show localized action in gastric mucosa, by providing high amount of drug at the site of action [107,108]. Nano formulations of antibiotics are another strategy that provide controlled delivery, enhanced bioavailability and decreased toxicity. The use of P-gp inhibitors in conjunction with antibiotics could be another approach to achieve sufficient concentration of antibiotic intracellularly [109]. In the following sections we discuss the possible formulation approaches that could be adopted to improve the bioavailability of drugs at the site of action, for the effective eradication of *H. pylori* infection. Various advantages of these novel formulations over the traditional formulations for the treatment of *H. pylori* infection are represented in Table 2, and the methods for fabrication of novel drug delivery system along with their specific advantages are presented in Supplementary Table S1.

**Table 2:** Reported novel drug delivery systems for the treatment of *H. pylori* infection.

Novel Drug Delivery Systems	Drug	Advantages over traditional formulation
Floating microspheres	Clarithromycin, Lisinopril	Floating microspheres increased the retention time of the formulation in the stomach, drug entrapment and prolonged release [110,111].
Floating beads	Amoxicillin trihydrate	pH sensitive amoxicillin trihydrate floating beads increased the retention time of the formulation in the stomach. It increased the bioavailability of drugs and eradicated *H. pylori* by more than 95% [112]
Floating pellets	Amoxicillin trihydrate	Increased drug release was observed, and 97% of drugs were released in 24 h [113].
Floating tablet	Clarithromycin, Amoxicillin trihydrate, Metronidazole, Levofloxacin	The tablet showed drug-loading ranging up to 81.7% and released the drug in a controlled manner (125 mg in 8 h and 250 mg in 12 h). Released drug for longer period of time. Metronidazole showed localized action with a low dose of drug [114,115].
Floating *in-situ* gel	Metronidazole	Reduced the dosing of metronidazole and released the drug sustainably and showed good efficacy in eradicating *H. pylori* [116].
Gastro retentive nano fibrous film	5-fluorouracil	It is a possible targeted therapy method for gastric cancer with better chemotherapeutic performance [117].
Gastro retentive wafer	Levofloxacin hydrochloride	It showed more efficacy against *H. pylori* due to retention of the formulation [118].
Mucoadhesive microspheres	Famotidine	Microspheres provided localized action of the drug, maximized drug release, and showed zero-order kinetics in drug release [119].
Gold NPs	Fruit extract of *Tribulus terrestris*	Showed bactericidal effect on the *H. pylori* bacteria [120].
Zinc oxide (ZnO) NPs	Metronidazole	ZnO-PEI NPs cause excessive formation of ROS within bacteria, resulting in cell membrane damage, RNA degradation, and consequent bacterial death [121].
Silver NPs	aqueous extract of *Toxicodendron vernicifuum*	It exhibited antibacterial action against *H. pylori* by inducing ROS, cell death, oxidative stress, and cytoplasmic DNA damage [122].
Bismuth NPs	Bismuth	Inhibited *H. Pylori* at concentrations ranging from 60 to 100 ng/ml. Bismuth nanoparticles have been seen to interact with the Krebs cycle, nucleotide metabolism, and amino acid metabolism, causing bacteria to produce metabolites such as uracil, glutamate, acetate, glycine, formic acid, valine, and glycine [123].
PLGA Nps	Clarithromycin	The MIC value of the NPs was 0.003 μg/ml to 0.1 μg/ml for the clinical *H. pylori* strain [124].
Polymeric NPs	Amoxicillin	It showed good efficiency for the *H. pylori* treatment and controlled release of drug at target site [125].
Lipid based NPs	Clarithromycin	It cures *H. pylori* infection by removing both biofilm and mucous layers blockage [126].

### 5.1 Gastroretentive drug delivery systems (GRDDS)

#### 5.1.1 Floating systems

**Fig. 2:**
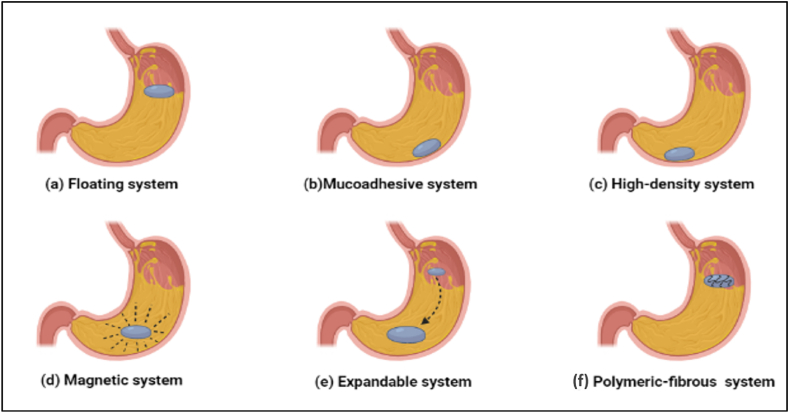
GRDDS systems for the management of *H. pylori* infection and *H. pylori* induced gastric ulcer.

The formulation is able to float in the gastric fluid due to the low density of floating systems, where it may stay for a longer period of time without changing the pace at which the stomach empties [127]. The low density is due to the swelling of polymer and gas generation. Polymer-based systems expand in the presence of gastric fluid and float in the juice [128]. The low-density gas generating system works by the production of carbon dioxide when it comes in contact with gastric fluids [129]. This system is suitable for *H. pylori* bacteria due to its localized action, which will directly deliver antibiotics in the gastric mucosa for longer periods. According to Javadzadeh et al. the floating system is suitable for medications that act locally on the stomach's gastric mucosa, such as metronidazole. Using this system, antimicrobial drugs can be applied topically to cure *H. pylori* infection, which may be helpful to prevent the adverse effects of traditional triple therapy [130]. Nilima et al. has developed floating gastroretentive beads of amoxicillin trihydrate, which inhibited the *H. pylori* growth effectively *in vitro* and *in vivo* [131]. Awasthi et al. developed the floating hollow micro balloons of amoxicillin, and an *in-vitro* investigation supported the proposed system's strong antibacterial efficacy. The hollow micro balloons loaded with amoxicillin appeared to be a good strategy for localized release of amoxicillin for the successful management of *H. pylori*-induced ulcers [132]. Kamsali et al. prepared a floating raft system of amoxicillin by guar gum and calcium carbonate to manage *H. pylori* infection. The optimized formulation showed better pharmacokinetics in comparison to marketed formulation and showed release of drug in controlled manner for more than 24 h [133]. The floating microspheres of clarithromycin developed by Tejaswi et al., to eradicate *H. pylori* bacteria showed 71% entrapment efficiency of the drug, and 82% of the microsphere floated for more than 12 h (11). Patel et al. and Emara et al., reported that the floating tablet of clarithromycin and amoxicillin has improved the prolonged release of the drug to the intestinal mucosa ([134,114]. A pictorial representation is provided in Fig. 2a for the floating GRDDS approach.

#### 5.1.2 Mucoadhesive systems

Bioadhesive polymers are used in this method so that they may stick to the stomach epithelial lining. Hydrophilic gelling chemicals coupled with several hydrogen-bond forming groups, such as sulfate, carboxyl, amide, and hydroxyl groups, are characteristic of macromolecular bioadhesive polymers. These include polycarbophil, carbopol, lectins, chitosan and gliadin etc. [135]. Villegas et al. developed a mucoadhesive system (mucolast) to eradicate *H. pylori* infection using amoxicillin and clarithromycin. Pharmacokinetics evaluation of the formulation showed more drug concentration on the stomach lining than in the systemic circulation. *In-vivo* efficiency of mucolast was done in *H. pylori* infected mice and showed significant results in terms of histopathological findings [136]. Dey et al. prepared floating mucoadhesive beads of amoxicillin trihydrate. The developed beads imparted good floating behavior for more than 24 h having a floating lag time of 46.3 ± 3.2 s(s). The optimized batch completely inhibited *H. pylori* development in *in vitro* culture after 15 h [137]. Patel et al. developed a mucoadhesive microsphere of amoxicillin-loaded carbopol-934P to eradicate *H. pylori* infection. The optimized formulation showed strong adhesion to the gastric mucosa. The drug entrapment was 80%, and the size was 109 μm. Microspheres released the drug in a sustained manner for more than 12 h [138]. Nagahara et al. reveled that using mucoadhesive microspheres containing amoxicillin to treat *H. pylori* infection in Mongolian gerbils infected with human *H. pylori* revealed that, when amoxicillin was provided as mucoadhesive microspheres instead of a suspension, it stayed in the stomach for longer. The anti-*H. pylori* effect of the amoxicillin-microspheres was higher than that of the amoxicillin suspension [139]. In a similar work, Adeola et al. assessed the efficacy of floating mucoadhesive beads *in vitro* against *H. pylori.* The study revealed that combining two gastro retentive systems has a synergistic effect in keeping the drug in the stomach for prolonged time. A floating and mucoadhesive system combination showed good *in vitro* results and localized action. Drug release from these beads was maintained via a mucin layer that was not disturbed, replicating the *in vivo* circumstances in which *H. pylori* is found in the gastric lining [140]. Umamaheshwari et al. reported the synergistic outcome of mucoadhesive nanoparticles of amoxicillin to treat *H. pylori* infection in the gerbil's model. The *in-vivo* evaluation of free drug amoxicillin and the mucoadhesive amoxicillin nanoparticles performed in the gerbil's model demonstrated superior anti-*H. pylori* effect of GRDDS [141]. A pictorial representation is provided in Fig. 2b for the mucoadhesive GRDDS approach.

#### 5.1.3 High-density systems

The density of the system is an essential feature for the stomach retention of the formulation. A high-density system uses its weight for the retention mechanism. When the system's density is higher than the gastric fluid, it will automatically descend to the stomach's bottom, placed below the pylorus. As a result of their resistance to the peristaltic contraction of the gastric wall, they eventually become imprisoned in the antrum. As a result, this system's stomach residency time is greatly extended. Commonly used density enhancers are iron, titanium dioxide, barium sulfate, zinc oxide powder, etc., increasing density by up to 1.5–2.4 g/ml [142]. Bera et al. revealed that the high-density system displays a considerably advanced *H. pylori* eradication rate than free medication [143]. Badhan et al. suggested that a high-density amoxicillin system can effectively treat *H. pylori* infection [144]. *Hao* et al. developed the sinking magnetic microparticles for the enhancement of gastric delivery of the antimicrobial agent. The optimized formulation showed *in-vitro* and *in-vivo* clearance of *H. pylori* infection [145]. A pictorial representation is provided in Fig. 2c for the high-density GRDDS approach.

#### 5.1.4 Magnetic systems

In this system, a dosage form is made of excipients, internal magnets, and active medicinal components. To control the location of the magnetic field, an extracorporeal magnet is placed over the stomach. The intensity produced by the extracorporeal magnet can affect gastro retention [146]. Some studies reported that the magnetic system increases the gastroprotection and bioavailability of the formulation [147,148]. This system has some drawbacks, like difficulty in precise positioning of the magnet and can result in low patient compliance. Silva-Freitas et al. developed magnetic polymeric stimulus-responsive particles for antimicrobial therapy in the stomach. The final composition of the microparticles was 9.0 ± 0.3% magnetite, 87.0 ± 2.3% Eudragit and 4.3 ± 1.5% amoxicillin. The optimized microparticles had a size of 17.2 ± 0.4 μm. It showed good anti-bacterial activity and magnetic field responsiveness. The magnetic field responsiveness of the particle has facilitated the antibiotic to enter a bacterial niche into the deep mucous layer. It also helped prevent unintended drug delivery into the acidic gastric lumen [149]. A pictorial representation is provided in Fig. 2d for the magnetic GRDDS approach.

#### 5.1.5 Expandable system

This system is designed to have a longer gastro retention time by increasing their volume or shape [150]. To function properly, an expandable system must meet three criteria: it must be small enough to be taken orally, in order to avoid passing through the pyloric sphincter, it must expand only in the stomach, and it must contract again after drug release is complete so that it can be expelled [151,152]. This formulation is also termed a “plug-type” system because it can block the pyloric sphincter. The system development occurs by the two methods, swelling and unfolding, allowing volume and shape modification [153,154]. Yang et al. fabricated a swellable asymmetric triple-layer tablet containing tetracycline, metronidazole, and bismuth for treating *H. pylori* that uses a floating feature to increase the retention period of drug in stomach. Tablets showed controlled release effect of tetracycline and metronidazole over 6–8 h [155]. Mahrouk et al. designed and optimized the metronidazole-loaded pH-sensitive hydrogel to treat *H. pylori* infection in animal models. The prepared hydrogel expanded by 700% in the first 4 h and released the medication throughout the course of 24 h. The hydrogel formula was more efficient against *H. pylori* than the widely existing oral metronidazole pills [156]. Kwak et al. prepared expandable beads using methylated sericin for the management of *H. pylori* infection [157]. Chang et al. prepared pH-sensitive hydrogel to deliver amoxicillin locally for *H. pylori* infection [158]. The floating in situ gel of amoxicillin developed by Patel et al. for treating *H. pylori*-based gastric ulcers showed a drug release of 95.78% up to 12 h and a swelling index of 67.16%, and concluded that it is a convenient candidate for treating *H. pylori*-based gastric ulcers [159]. A pictorial representation is provided in Fig. 2e for the expendable GRDDS approach.

### 5.2 Nanomedicine

**Fig. 3:**
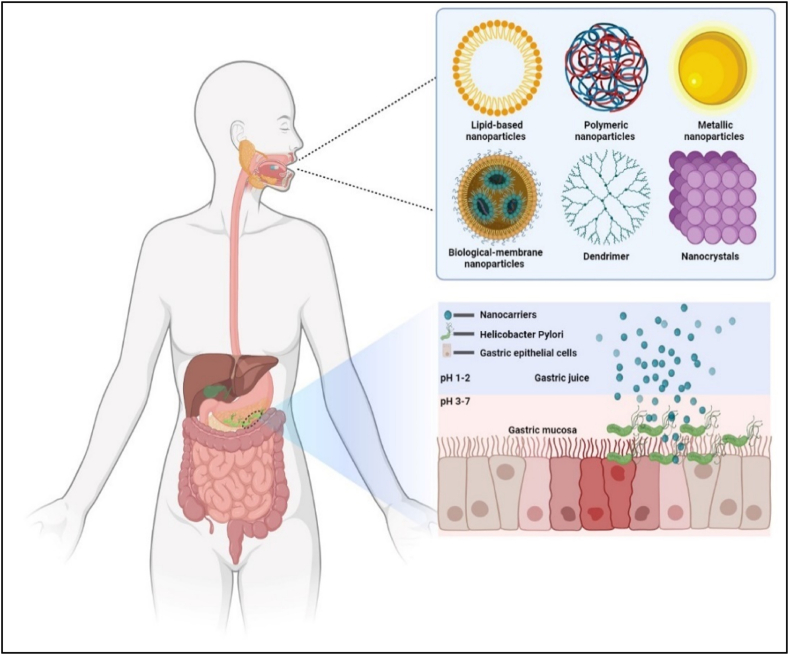
Pictorial representation of available nanoformulation for the management of *H. pylori* infection.

The Nobel laureate Richard P. Feyman first introduced nanotechnology in his lecture ‘There's Plenty of Room at the Bottom’ in 1959 [160]. Nanotechnology is well known strategy for the delivery of drug since the last century. Nanoparticles (NPs) are tiny particles with size ranges from 1 to 1000 nm. Nanoparticles include liposomes, lipid NPs, polymeric NPs, membrane NPs, metal NPs, nanocrystals, ceramic NPs, etc. Enhanced biological activity of NPs is largely due to their increased surface area and compactness [124]. There are two separate mechanisms by which they engage the targeted cells. First, NPs bind to the cell membrane and transport the medication. Second, the NPs penetrate the cell membrane and start releasing the drug from inside [124,161]. The good drug entrapment and targeting ability of nano formulation makes it an effective option for treating *H. pylori* infection and *H. pylori-*based ulcer. NPs are shown to possess anti-bacterial activity through various mechanisms such as the production of reactive oxygen species (ROS) or the disruption of cellular membranes, genetic components, or proteins [[162], [161]]. It is also reported that NPs can protect the drug degradation from the acid and enzymes in the gastric environment. NPs also show localized action and controlled release of drug at the target site. Drug-loaded NPs achieve their therapeutic effect via localized accumulation and sustained release. This postulates that the nanoparticle-based formulation of antibiotics is a good option for treating *H. pylori* [[163], [164], [165]]. Furthermore, when compared to direct antibiotic use, nanoparticular systems have imparted less toxicity in therapy, and these strategies play critical part in reducing antibiotic resistance. Fig. 3 displays several types of NPs for the management of *H. pylori* infection.

#### 5.2.1 Metallic nanoparticles

**Fig. 4:**
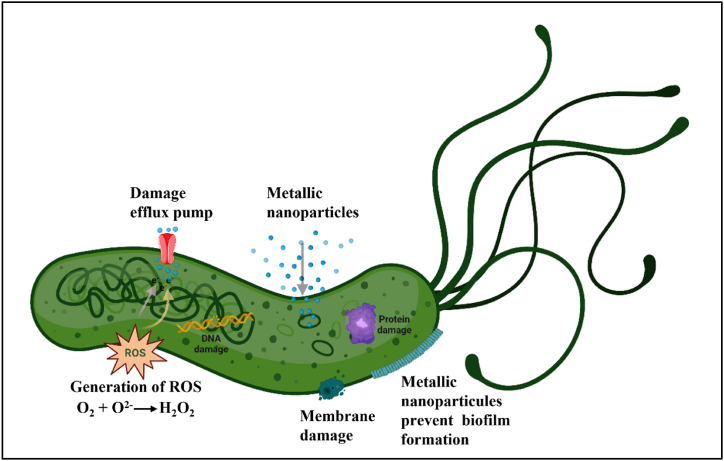
Metallic nanoparticles targeting *H. pylori* infection.

Metallic nanoparticles are fabricated from pure metal particles such as zinc, gold, and silver, which have their own distinct surface charges and hydrophobicity [166]. This facilitates the nanoparticle to target with plasma proteins, the immune system, matrices, disseminating and therapeutic substances throughout the biological system [167]. It is reported that nanoparticles made of bismuth, zinc, gold, and silver to treat *H. pylori* were effective by inducing inflammation, oxidative stress, apoptosis, and biofilm inhibition [168]. Gopinath et al. revealed that, gold NPs using the fruit extract of *Tribulus terrestris* showed bactericidal effect on the *H. pylori* bacteria [120]. Chakraborti et al. designed the 3–7 nm Zinc oxide (ZnO) NPs coupled with polyethyleneimine (PEI) to prevent the growth of a strain of *H. pylori* resistant to the antibiotic metronidazole. This ZnO-PEI NPs caused excessive formation of ROS within bacteria, resulting in cell membrane damage, RNA degradation, and consequent bacterial death. This demonstrates that antibiotics may be safely employed with ZnO-PEI NP at concentrations below those at which they cause damage to human cells [121]. Saravanakumar et al. synthesised silver (NPs) with a mean particle size of 2–4 nm by employing an aqueous extract of *Toxicodendron vernicifuum*. It exhibited antibacterial action against *H. pylori* by inducing ROS, cell death, oxidative stress, and cytoplasmic DNA damage [122]. ZnO NPs are safe according to the US Food and Drug Administration [169]. Bismuth NPs such as ranitidine bismuth citrate (RBC) and colloidal bismuth subcitrate (CBS), which are extensively used for the management of gastrointestinal diseases including *H. pylori* infection in combination with antibiotics [170]. Nazari et al. reported that Bismuth nanoparticles inhibited *H. pylori* at concentrations ranging from 60 to 100 ng/ml. Bismuth nanoparticles have been seen to interact with the Krebs cycle, nucleotide metabolism, and amino acid metabolism, causing bacteria to produce metabolites such as uracil, glutamate, acetate, glycine, formic acid, valine, and glycine [123]. Fig. 4 presents a pictorial representation of the metallic nanoparticles' antimicrobial mechanism.

#### 5.2.2 Polymeric nanoparticles

Polymeric NPs are biodegradable organic NPs with nano-spherical or nano-capsular forms [171]. Due to their high drug loading ability and stability, polymeric NPs are widely employed in drug-delivery system [172,173]. These NPs are favoured in therapeutic techniques because they offer benefits such as high endocytosis capacity, active or passive drug targeting, and high drug encapsulation [[174], [175], [176]]. Polymeric NPs are gaining attention to manage many pathogenic infections including *H. pylori.* Polyacrylic acid, poly lactic-*co*-glycolic acid (PLGA), alginate, albumin, gliadin, chitosan, and cellulose are the most extensively utilized polymers to make polymeric nanoparticles for fighting *H. pylori* due to their pH sensitivity, mucosal adhesiveness, and biocompatibility [166,[177], [178], [179], [180]]. PLGA NPs are the most widely utilized polymeric NPs. PLGA nanoparticles have less toxicity and are FDA approved [181]. Lutfipour et al. developed clarithromycin encapsulated PLGA NPs and tested on clinical strains of *H. pylori*. The MIC value of the NPs was 0.003 μg/ml to 0.1 μg/ml [124]. One more good example of mucoadhesive polymer is polyethyl-cyanoacrylate (PECA). The mucoadhesive nature of PECA offers significant prospects for developing *H. pylori* medication delivery systems [182]. Harsha et al. produced carbopol nanospheres based on their mucoadhesive characteristic to sustainably and efficiently release amoxicillin in the stomach in a *H. pylori* therapy [183]. Umamaheshwari et al. prepared PECA based lectin-modified gliadin NPs, which deliver the acetohydroxamic acid and block the binding of several *H. pylori* bacteria to gastric mucosal cells [184]. Lectin-modified gliadin NPs were employed to transport amoxicillin, clarithromycin, and omeprazole simultaneously, achieving a 16% higher eradication rate against *H. pylori* than the standard triple treatment [185]. A pictorial representation is provided in Fig. 2f for the polymeric fibrous approach.

#### 5.2.3 Targeting nanoparticles

**Fig. 5:**
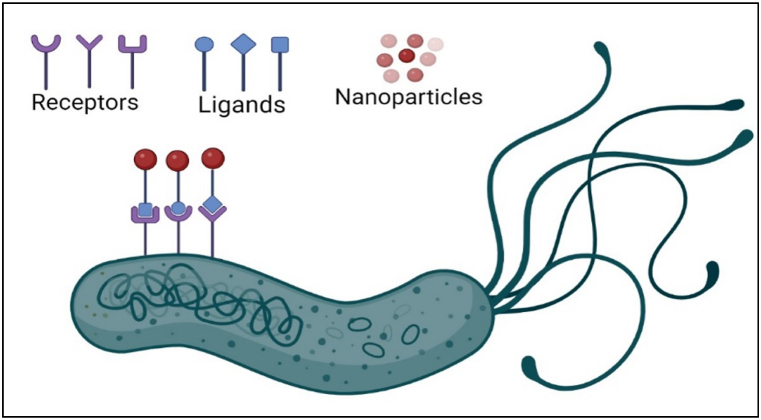
Surface targeting nanoparticles for the treatment *H. pylori* infection.

**Fig. 6:**
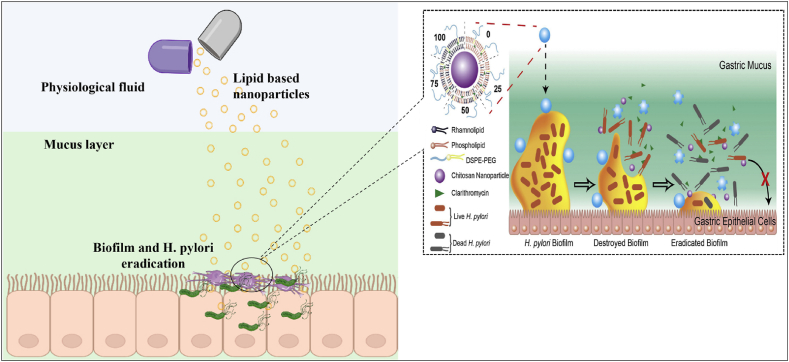
Surface modified Lipid based nanoparticles (LPNs) for the eradication of *H. pylori*.

To ensure that the drug-carrying NPs will deliver drug to their intended target site, their surfaces are modified by the addition of suitable surface atoms, organic groups, or targeting receptors [186]. Surface modification can help to increase the stability of NPs, improves interaction and aqueous solubility [187]. Many preliminary works have been done in this area by modifying NPs to target *H. pylori*. The stomach's acidic environment makes it challenging to eradicate *H. pylori* due to the insufficient reach of antibiotics in the affected region. To get over this problem, scientists have developed a urea-mediated targeted drug delivery system consisting of pH-sensitive urea-modified UCCs- 2-PLGA NPs. These NPs have been found to have greater anti-*H. pylori* action, lesser cytotoxicity, more specificity, and improved pH sensitivity. It is reported by Chang et al. that amoxicillin-loaded genipin cross-linked fucose-chitosan will interface with *H. pylori* and show efficient and precisely controlled release [125]. Yu-hsin lin et al. examined the efficiency of berberine-loaded fucose receptor-linked chitosan NPs against *H. pylori in-vivo* at a dosage of 6 mg/L on C57BL/6 mice, which resulted in 40% inhibition [188]. The results suggest that NPs containing targeting receptors efficiently eradicate *H. pylori*. Adding ligands or targeting molecules designed to interact with bacteria to the surface of the NPs enable selective targeting of the drug carrier NPs to the target areas [189]. Fig. 5 is a schematic diagram that illustrates the interactions between the targeted NPs surfaces during *H. pylori* treatment (see Fig. 6).

#### 5.2.4 Lipid nanoparticles

Lipid nanoparticles have been widely employed as medication carriers and chemical agent delivery in biological applications. The lipid NPs have a solid lipid core surrounded by a matrix of soluble lipophilic molecules, and their sizes range from 10 to 1000 nm. Surfactants and emulsifiers were used to maintain the exterior's structural integrity [190]. Liposomes are spherical in morphology and include aqueous-core vesicles composed of phospholipids and other lipids in the form of one or more bilayers. Lipid components like cholesterol and quaternary ammonium have been incorporated in the lipid bilayer, where cholesterol reduces lipid bilayer permeability and promotes membrane stability and fluidity [191]. It is essential to generate adhesion through receptor in the gastric epithelium for *H. pylori* and suppress outer membrane proteins, which can minimize bacterial cell colonization and infection. Arif et al. prepared the lipid polymer nanoparticles, which has the property to combine with the biofilm of bacteria and get dissolved in the biofilm, delivering the drug directly into the bacterial cells [192]. Recent research suggests that amoxicillin-encapsulated pectin liposomes have anti-adhesive properties for *H. pylori* [193,194]. Lipid NPs have demonstrated versatility, making them an effective substrate for administering diverse antimicrobial drugs. Furthermore, the benefits of triple therapy in combination with linolenic acid on the mice GI microbiota were investigated. The findings revealed that triple treatment generated considerable alterations compared to linolenic acid, which had a minimal effect. This suggests that liposomal linolenic acid therapy is efficacious and safe for eradicating *H. pylori* and reducing *H. pylori* colonization. Saraf et al. developed a nanostructured lipid carrier of Hesperidin (Hesp) and clarithromycin for *in vitro H. pylori* eradication. The outcomes showed that NLCs made interaction with the bacterial call wall by sticking to its outer cell wall without harming the human cells and then disrupting the membrane, resulting in cytoplasmic leakage.

**Fig. 7:**
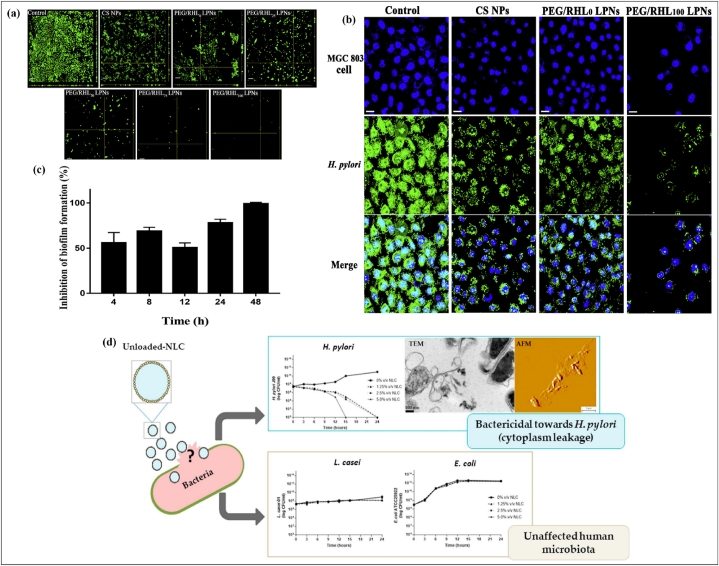
Reported activity of LPNs and NLCs against *H. pylori* (a) Comparison of biofilm inhibition activity of polymeric and LPNs with control (b) Hoechst and SYSTO9 staining data demonstrating the adhesion inhibition ability of LPNs on the *H. pylori* and MGC803 biotic cells (c) Time-dependent inhibitory effect of PEG/RHL100 LPNs on biofilm formation (d) Anti *H. pylori* activity of NLC by altering cell wall fluidity at small doses without affecting other gut bacteria (such as *L. casei* and *E. coli*). Figures reproduced with permission [126,196].

Furthermore, their findings revealed that NLCs had a synergistic impact on *H. pylori*, which might be owing to the Hesp component causing harm to the microbial lipid layer, increasing the permeability of the antibiotic to the bacterial cell [195]. Further, Sharaf and colleagues showed that a combination of NLCs and magnetic NPs loaded with Amoxicillin and Clarithromycin demonstrated enhanced efficiency for *H. pylori* and a reduction of drug side effects on normal cells. The mechanism of lipid-based NPs (LPNs) for the eradication of *H. pylori is* shown in Fig. 7. Li et al. discovered novel clarithromycin LPNs to cure *H. pylori* infection by removing both biofilm and mucous layers blockage. Chitosan nanoparticles (CS NPs) were used in LPNs as the exterior, mixed lipid layers comprising rhamnolipids (RHL) as the center, and DSPE-PEG2000 was added to the surface of LPNs to enhance hydrophilicity. The *H. pylori* biofilm was totally eradicated by the formulated LPNs. PEGylated LPNs quickly pass through mucus and efficiently remove the *H. pylori* biofilm that was embedded in the mucus layer without reacting with mucins Fig. 7a. Staining data demonstrating the adhesion inhibition ability of LPNs on the *H. pylori* as well as MGC803 biotic cells is demonstrated in Fig. 7b. The time-dependent inhibitory effect of PEG/RHL100 LPNs on biofilm formation is shown in Fig. 7c [126]. Seabra et al. discovered that *H. pylori* can be eliminated, even at small concentrations, using nanostructured lipid transporters that are not loaded with medicines. Cultures of *H. pylori* with nanostructured lipid transporters (1.25%) were examined using microscopic techniques, which revealed that after 12 h the membranes of most of the bacteria had been broken. This demonstrated that the *H. pylori* barrier can be destroyed by the nanostructured lipid transporters. The capacity of nanostructured lipid transporters to be kill *H. pylori* while having no effect on intestinal flora has been shown Fig. 7d [196].

### 5.3 P-glycoprotein inhibitors

**Fig. 8:**
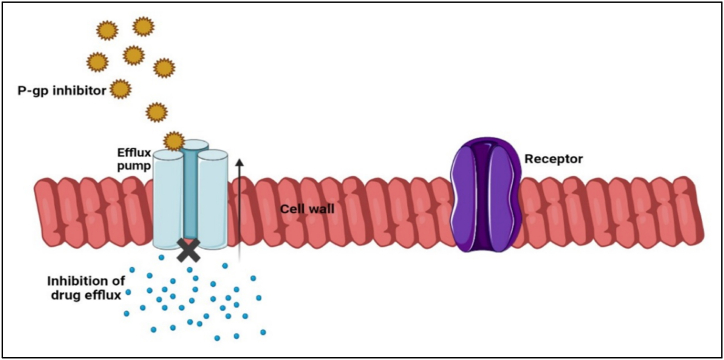
P-gp inhibitors preventing the drug efflux.

P-glycoprotein (P-gp) is a transmembrane glycoprotein (170 kDa) that comes under a broad superfamily of ATP-binding cassette (ABC) transporters [197,198]. P-gp influences the absorption and permeability of drug molecules and is being widely used as a bioavailability booster [199,200]. A study conducted in the Australian population reveled that the increase in P-gp level resulted in drug resistance towards *H. pylori* [93,199]. Damanhuri et al. reported the effect of p-gp levels in the drug absorption and for treatment of *H. pylori* infection. They observed a dramatic increase in the *H. pylori* induced ulcer development in the P-gp-expressed rats compared to the P-gp inhibited ones [201]. Babic et al. performed research to check the involvement of P-gp inhibitors in the multidrug resistant *H. pylori* infection. The results revealed that the P-gp inhibitors effectively eradicated *H. pylori* infection in 20 out of 33 patients treated with p-gp inhibitors [202]. Omar et al. evaluated the p-gp level in *H. pylori* patients to understand the effect of P-gp on treatment failure in *H. pylori* infection. In comparison with the group that were *H. pylori*-negative, the resistant group had greater relative antral P-gp expression (*p* < 0.0361), and the majority of individuals showed resistance towards clarithromycin (72%), metronidazole (63.6%), or both (54.5%) [203]. Omar et al. determined the efficacy of P-gp on antibiotic resistance for the treatment of *H. pylori* infection. The result showed that P-gp expression differed significantly between *H. pylori*-positive and *H. pylori*-negative individuals (*P* < 0.028). In antral specimens, the TT genotype showed substantially less P-gp activation than the CC genotype for the MDR1 C3435T polymorphism (*p* < 0.041). P-gp activation was also substantially different in the homozygous TT genotype with *H. pylori* infection in comparison to *H. pylori*-negative individuals (*p* < 0.029) [204]. All these data suggests that P-gp inhibitor can give a synergistic effect in combination with antibiotics and PPIs for treatment of *H. pylori*. The mechanism of P-gp inhibitor to stop the drug efflux shown in Fig. 8.

## 6 Conclusions

Treatment of *H. pylori* infection and associated gastric ulcer is always a concern for the treating doctor. The current therapeutic regimens are not very effective due to antibiotic resistance and patient noncompliance. Development of a new formulation is urgently required to overcome the challenges of the current modalities. As discussed in this review, strategies like GRDDS, p-gp inhibitors, and nano-formulations have proved to be useful *in vitro* as well as in a few animal models. After the comprehensive review of the various approaches to overcome the challenges, authors recommend here a strategy for effective eradication of *H. pylori* infection and the associated gastric ulcer. We recommend a nano-formulation of antibiotics incorporated as a GRDDS system for eradication of *H. pylori* infection. The treatment regimen should include a P-gp inhibitor and a PPI along with the GRDDS system containing the antibiotic. The proposed novel formulation is expected to significantly improve the treatment efficacy as it can allow precise target specific drug binding. The formulation is expected to provides better stability for antibiotics from the acidic environment and assures elevated local drug concentration. By design, GRDDS extends the time duration of the drug availability in the stomach. This extension in contact time facilitates sustained interactions with bacterial colonies, substantially enhancing the probability of successful eradication. The advantages of employing gastroretentive formulations encompass elevated bioavailability of medications, reduced dosing frequency, and potentially improved patient compliance in the treatment regimen. As indicated in the literature, the mucoadhesive approach appears to offer better convenience and efficacy in patients compared to other GRDDS in the context of *H. pylori* infection treatment. The mucoadhesive system exhibits the capability to adhere to the mucosal surface, enabling direct and targeted contact with the bacteria. This direct interaction holds the potential of significantly enhancing the efficacy of *H. pylori* infection treatment. The nano-formulation owing to its diminutive scale, increased surface area, and efficient compactness offer an additional layer of advancement by the encapsulation of antibiotics thereby providing prolonged release and increased bioavailability and minimized side effects. The extended-release property of nano-formation enhances the antibiotic's effectiveness and could potentially reduce the frequency of dosing and improves patient compliance. Leveraging polymeric nanoparticles within the framework of GRDDS presents a potent strategy against *H. pylori* infection. Both floating and mucoadhesive GRDDS systems demonstrate compatibility with nano-formulations, showcasing their suitability for such therapeutic endeavour. Phytochemicals and natural compounds, when used as P-gp inhibitors in conjunction with standard antibiotics, prevent their efflux and can improve the treatment outcomes, offering a more comprehensive therapeutic approach. Integrating nanoparticles into a GRDDS alongside a P-gp inhibitor and PPI holds the potential of novel approach for eradication of *H. pylori* infections. Concurrently, the intrinsic *H. pylori* eradication property of the PPI contributes to the formula's effectiveness. The inclusion of a P-gp inhibitor not only eradicates biofilm formations but also impedes drug efflux mechanisms, further boosting the treatment strategy. This multifaceted approach has the capacity to redefine the landscape of *H. pylori* infection management. Thus, a complete eradication of *H. pylori* could be achieved with this innovative approach by assuring sufficient drug concentrations locally and systemically for an extended period of time, which would also prevent the development of resistance to antibiotics.

## 7 Challenges and future prospects

As previously mentioned, novel formulations offer numerous advantages; however, the formulation development process is not without its challenges. These include the need to select suitable antibiotics and excipients based on compatibility, ensuring drug stability in gastric juices, optimizing the drug-polymer ratio to prolong stomach retention, determining the appropriate drug release kinetics, addressing cost and treatment accessibility, and successfully translating the formulation into clinical applications.

Conducting in-vivo evaluations of the formulation presents another significant challenge due to the limited availability of facilities for culturing *H. pylori* bacteria. These bacteria are anaerobic in nature, demanding specialized growth conditions and specific media for cultivation. Additionally, identifying a suitable animal model that accurately replicates the conditions of the human stomach for *H. pylori* infection poses its own set of considerable challenges. Handling *H. pylori* poses a big challenge as the bacteria has a high contamination rate. Antibiotic formulations may be less effective since *H. pylori* has shown increased resistance to these drugs by genetic modifications. Additional difficulties arise when attempting to assess the formulation's effectiveness within the context of combination therapy. Reproducibility of the experimental results in an animal model is also challenging.

## Funding

The work is funded by 10.13039/501100001411Indian Council of Medical Research, New Delhi, India. **File no.** 3/2/2/16/2022-NCD-III.

## CRediT authorship contribution statement

**Ashutosh Gupta:** Writing – review & editing, Writing – original draft, Visualization, Methodology, Funding acquisition, Formal analysis, Data curation, Conceptualization. **Shiran Shetty:** Writing – review & editing, Supervision, Formal analysis, Data curation, Conceptualization. **Srinivas Mutalik:** Writing – review & editing, Supervision, Investigation, Formal analysis, Data curation, Conceptualization. **Raghu Chandrashekar H:** Writing – review & editing, Supervision, Investigation, Conceptualization. **Nandakumar K:** Writing – review & editing, Supervision, Methodology, Data curation, Conceptualization. **Elizabeth Mary Mathew:** Conceptualization, Writing – review & editing. **Abhishek Jha:** Writing – original draft, Data curation, Conceptualization. **Brahmeshwar Mishra:** Writing – review & editing, Supervision, Conceptualization. **Siddheesh Rajpurohit:** Writing – review & editing. **Gundawar Ravi:** Writing – review & editing. **Moumita Saha:** Writing – review & editing. **Sudheer Moorkoth:** Writing – review & editing, Visualization, Supervision, Resources, Investigation, Data curation, Conceptualization.

## Declaration of competing interest

The authors declare that they have no known competing financial interests or personal relationships that could have appeared to influence the work reported in this paper.
