# Supplementary material for: Treatment of H. pylori infection and gastric ulcer: Need for novel Pharmaceutical formulation
Source: Heliyon. 2023 Sep 24;9(10):e20406. doi: 10.1016/j.heliyon.2023.e20406 (PMC10550623; doi:10.1016/j.heliyon.2023.e20406)
Supplement: Multimedia component 1 [file mmc1.docx]

Supplementary Table S1. Fabrication method for the novel drug delivery system

| **S.No.** | **Formulation name** | **Method used** | **Procedure** | **Advantage** | **Disadvantage** |
| --- | --- | --- | --- | --- | --- |
| 1 | Floating microspheres [1] | Solvent diffusion evaporation technique | The drug and polymer are dissolved in a suitable organic solvent. The organic solution is slowly added into the aqueous solution of PVP or PVA. Organic solvent will be evaporated using continuous stirring for 3 h at ambient temperature. Prepared microcapsules were washed with distilled water and dried at ambient temperature. | - Prolonged Gastric Residence Time - Enhanced Drug Delivery - Reduced frequency of dosing - Protection of acid-labile drugs | - Gastric emptying variability - Limited drug capability - Gastric irritation |
| 2 | Mucoadhesive beads [2] | Ionotropic gelation method | The sodium alginate will be added in double distilled water with stirring at 1000 rpm. The drug will added in the polymeric solution and allowedfor proper mixing on a stirrer. Separately, prepare the calcium chloride solution and polymeric solution. Add the above solutions into the drug solution using an 18G syringe. Filter the prepared beads and wash with distilled water and dry them in the oven. | - Enhanced drug retention - Localized action - Reduced systemic side effects | - Potential irritation - Mucus barrier variability - Gastrointestinal clearance |
| 3 | Floating matrix tablet [3] | Compression method | Weigh and mix all the pharmaceutical ingredients, APIs and excipients. Prepare the damp mass by adding solvent to the powder mix. This is followed by preparing the granules from the damp mass and compressing the granules in a tablet punching machine. | - Prolong gastric residence time - Improved drug release profile - Uniform drug distribution - Ease of administration | - Gastric emptying variability - Gastrointestinal clearance |
| 4 | Expendable system [4] | Hot Melt Extrusion | Prepare the drug-polymer bland by mixing drug-polymer and a suitable solvent. The bland is dried at 40 ℃ and then grounded by a blade mill. Hot melt extrusion technique is further used to prepare the formulation. | - Gastric retention - Sustained drug release - Ease of administration | - Risk of burst release of drug - Gastrointestinal obstruction |
| 5 | Magnetic microparticles [5] | Precipitation method | First prepare the magnetic nanoparticles by adding the metallic compounds in a suitable solvent. Allow the reaction to happen and precipitate to form. The p precipitated particles are further washed with ethanol. The prepared nanoparticle are further loaded in the emulsion for further use. | - A magnetic field controls gastric permanence time. - Adaptation with magnetic hyperthermia as an additional antibacterial mechanism | - External magnetic field makes this system inconvenient, - Low patient compliance. - Low specificity |
| 6 | High density system [6] | Compression method | Pass all the excipients through the 80 mesh. Then prepare the granules of the excipients and drug. Press the granules into tablets using the tablet punching machine. | - Deposition in antrum rugae or folds, increases gastroretention for long periods. - Feed state and the presence of fluid in the stomach do not influence gastroretention | - lack of clinical and animal studies. - Technically difficult to produce - Contains significant amounts of drugs. - Sedimentation can cause gastric irritation |
| 7 | Liposomes [7] | solvent vaporization | The lipids will be weighed and mixed in a suitable organic solvent. Then organic solventwill then be evaporated using a rota evaporator. | - Biocompatibility - Low toxicity - Selectivity - Side effects reduction - Biodegradable - Excellent applicability - Versatility in incorporating - different bioactive agents - Drug protection against external environment degradation | - Stability can be impaired by the action of chemical, physical and biological components, shortening its half-life. Parameters that influence this system: alteration of the vesicle, leakage of constituents, exposure of the drug into the external environment and recognition by the complement system and macrophages |
| 8 | Nano emulsion [8] | water-in-oil emulsification | Mix the oil phase with a suitable surfactant. Separately mix the aqueous phase with a co-surfactant. Add one phase to another phase in a dropwise manner with continuous stirring. | - Better bioavailability - Greater action against infection Greater selectivity Reduction of adverse effects Drugs protection against immune system responses and degradation Lower dose for better efficacy | - Use of surfactants that can be cytotoxic - Low stability |
| 9 | Polymeric nanoparticle [9] | Polymeric dispersion | Add polymeric solution to the organic solution stirring. After precipitation the particle was washed with water and dried at 40 ℃ | - Modulation of the particle diameter - Long-term stability - Biocompatibility - Biodegradability - Ability to incorporate lipophilic and hydrophilic compounds | - May have low physical chemical stability. - Nanoparticles aggregation |
| 10 | Metallic nanoparticles [10,11] | Evaporation-condensation method | To make a metal precursor solution, dissolve the right metal salt in the right solvent. Nanoparticle size and concentration are also affected by the metal salt concentration. The metal precursor solution should be stabilised using suitable agents. During synthesis, the stabilising agent aids in regulating the nanoparticles' size and stability. Stir the metal precursor solution while adding the reducing agent. The metal ions are converted into metallic nanoparticles when an electron is donated by the reducing agent. To speed up the reduction process and exert more precise control over the nanoparticle size, it may be necessary to heat or reflux the reaction mixture. As the metallic nanoparticles are created, the colour of the solution will shift throughout the synthesis. | - Low toxicity - Chemical stability - Adjustable pore size - Easy decomposition - Reduced adverse effects - Catalytic activity - UV photoactivity - Greater antimicrobial activity | - Difficulties in the synthesis process - High material cost - Nanoparticles |

**Reference**

[1] N.F. El Nashar, A.A. Donia, O.Y. Mady, G.M. El Maghraby, Formulation of clarithromycin floating microspheres for eradication of Helicobacter pylori, J Drug Deliv Sci Technol. 41 (2017) 213–221. https://doi.org/10.1016/j.jddst.2017.07.016.

[2] N.A. Thombre, P.S. Gide, Floating-bioadhesive gastroretentive Caesalpinia pulcherrima-based beads of amoxicillin trihydrate for Helicobacter pylori eradication, Drug Deliv. 23 (2016) 405–419. https://doi.org/10.3109/10717544.2014.916766.

[3] S. Ahmad, J.A. Khan, T.N. Kausar, M.H. Mahnashi, A. Alasiri, A.A. Alqahtani, T.S. Alqahtani, I.A. Walbi, O.M. Alshehri, O.A. Elnoubi, F. Mahmood, A. Sadiq, Preparation, Characterization and Evaluation of Flavonolignan Silymarin Effervescent Floating Matrix Tablets for Enhanced Oral Bioavailability, Molecules. 28 (2023). https://doi.org/10.3390/molecules28062606.

[4] A. Melocchi, M. Uboldi, N. Inverardi, F. Briatico-Vangosa, F. Baldi, S. Pandini, G. Scalet, F. Auricchio, M. Cerea, A. Foppoli, A. Maroni, L. Zema, A. Gazzaniga, Expandable drug delivery system for gastric retention based on shape memory polymers: Development via 4D printing and extrusion, Int J Pharm. 571 (2019). https://doi.org/10.1016/j.ijpharm.2019.118700.

[5] S. Hao, Y. Wang, B. Wang, Sinking-magnetic microparticles prepared by the electrospray method for enhanced gastric antimicrobial delivery, Mol Pharm. 11 (2014) 1640–1650. https://doi.org/10.1021/mp5000339.

[6] J. Guan, L. Zhou, S. Nie, T. Yan, X. Tang, W. Pan, A novel gastric-resident osmotic pump tablet: In vitro and in vivo evaluation, Int J Pharm. 383 (2010) 30–36. https://doi.org/10.1016/j.ijpharm.2009.08.043.

[7] S. Thamphiwatana, W. Gao, M. Obonyo, L. Zhang, In vivo treatment of Helicobacter pylori infection with liposomal linolenic acid reduces colonization andameliorates inflammation, Proc Natl Acad Sci U S A. 111 (2014) 17600–17605. https://doi.org/10.1073/pnas.1418230111.

[8] Y.H. Lin, S.F. Chiou, C.H. Lai, S.C. Tsai, C.W. Chou, S.F. Peng, Z.S. He, Formulation and evaluation of water-in-oil amoxicillin-loaded nanoemulsions using for Helicobacter pylori eradication, Process Biochemistry. 47 (2012) 1469–1478. https://doi.org/10.1016/j.procbio.2012.05.019.

[9] D. Luo, J. Guo, F. Wang, J. Sun, G. Li, X. Cheng, M. Chang, X. Yan, Preparation and evaluation of anti-helicobacter pylori efficacy of chitosan nanoparticles in vitro and in vivo, J Biomater Sci Polym Ed. 20 (2009) 1587–1596. https://doi.org/10.1163/092050609X12464345137685.

[10] S. Chakraborti, S. Bhattacharya, R. Chowdhury, P. Chakrabarti, The Molecular Basis of Inactivation of Metronidazole-Resistant Helicobacter pylori Using Polyethyleneimine Functionalized Zinc Oxide Nanoparticles, PLoS One. 8 (2013). https://doi.org/10.1371/journal.pone.0070776.

[11] V. Gopinath, S. Priyadarshini, D. MubarakAli, M.F. Loke, N. Thajuddin, N.S. Alharbi, T. Yadavalli, M. Alagiri, J. Vadivelu, Anti-Helicobacter pylori, cytotoxicity and catalytic activity of biosynthesized gold nanoparticles: Multifaceted application, Arabian Journal of Chemistry. 12 (2019) 33–40. https://doi.org/10.1016/j.arabjc.2016.02.005.
